# Lichenoid Skin Nodules as Presenting Feature of Necrotic Erythema Nodosum Leprosum in Leprosy

**DOI:** 10.4269/ajtmh.20-0177

**Published:** 2020-07-08

**Authors:** Akanksha Kaushik, Sunil Dogra, Tarun Narang

**Affiliations:** Department of Dermatology, Venereology and Leprology, Postgraduate Institute of Medical Education and Research, Chandigarh, India

**Figure 1.**
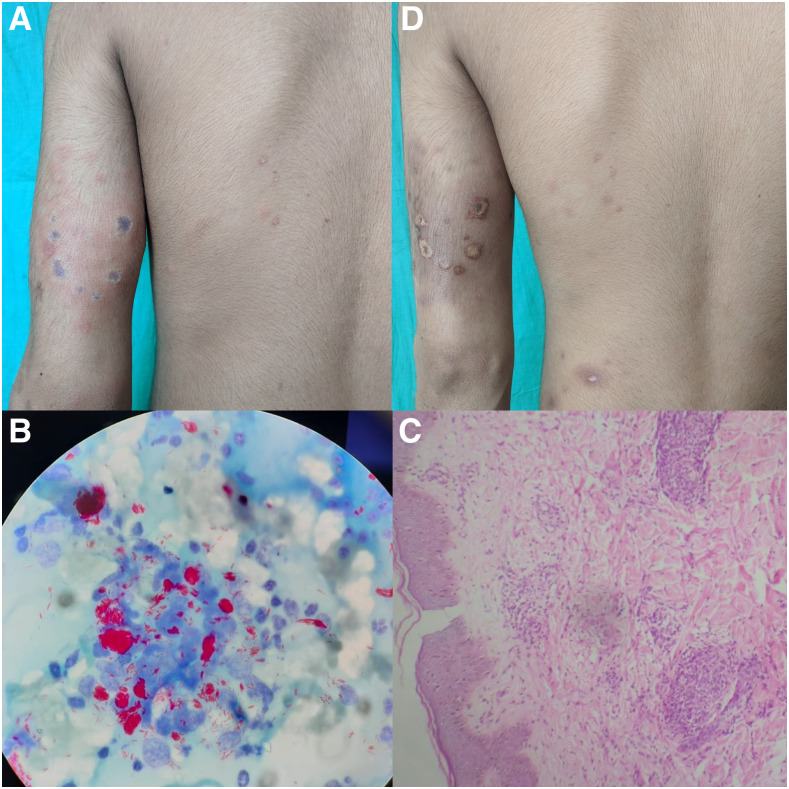
(**A**) Patient with multiple papulo-nodules, having a lichenoid center with surrounding blisters, on upper limb and back. (**B**) Slit skin smear of the same patient showing acid fast bacilli. (**C**) Histopathological examination of skin biopsy showing granulomatous inflammation and vasculitis. (**D**) Same patient with ulcerative changes in lesions within 2 weeks, suggesting necrotic ENL. This figure appears in color at www.ajtmh.org.

An 18-year-old male presented with painful papulo-nodular lesions on the upper limbs and back along with high-grade fever since 1 week. Most lesions had a lichenoid center with surrounding blisters (Figure 1A). There was associated tenderness and thickening of bilateral ulnar nerves. Slit skin smear was positive for *Mycobacterium leprae* (with a bacteriological index of 6+ and a morphological index of 0%) (Figure 1B). Skin biopsy revealed features of lepromatous leprosy (LL) with erythema nodosum leprosum (ENL) including granulomas, nerve destruction, vasculitis, and panniculitis, and lepra stain was positive (Figure 1C). Over the next 2 weeks, most lesions developed ulceration, suggesting necrotic ENL (Figure 1D). The patient is currently receiving WHO-recommended multidrug therapy (MDT) with oral corticosteroids.

Erythema nodosum leprosum is a type III hypersensitivity reaction presenting as erythematous, tender papulo-nodules, commonly on extremities, with histology showing granulomatous inflammation with neutrophil infiltration, and variable degrees of panniculitis and vasculitis.^1,2^ Reported incidence of ENL in multibacillary leprosy (especially LL) is as high as 11.8%.^3^ The lesions usually appear after starting MDT, although it may sometimes be the presenting feature.^4^ Rarely, ENL may be associated with ulceration, producing necrotic ENL, also called erythema necroticans. Necrotic ENL is a severe form often associated with systemic complications and heals with scarring.^5^ The case illustrates that lichenoid skin nodules may be an unusual initial presentation of necrotic ENL.
